# Supplementary material for: The influence of the solid to plasma phase transition on the generation of plasma instabilities
Source: Nat Commun. 2017 Nov 23;8:1713. doi: 10.1038/s41467-017-02000-6 (PMC5700939; doi:10.1038/s41467-017-02000-6)
Supplement: Supplementary file 1 — Supplementary Information [file 41467_2017_2000_MOESM1_ESM.pdf]

Supplementary Note 1 | Numerical modelling and simulations

In this work, two basic simulation approaches describe in full detail the dynamic evolution of matter into plasma. We adopt Finite Element Method (FEM) and Magneto-Hydrodynamic (MHD) numerical schemes to model the transition of a metal wire from the solid to the plasma phase. The 3D FEM simulations are performed using the commercial FEM code LS-DYNA[1,2]. The developed FEM approach combines the electromagnetic (EM), thermal, structural and hydrodynamic equations in a single coupled multiphysics model. The electromagnetic FEM coupling allows for the application of electrical currents on the wire to compute the associated magnetic and electric fields and determine the induced currents. The evolution of the electromagnetic fields is computed by solving the Maxwell equations using the Eddy-current (induction-diffusion) approximation[2]. The solution of these coupled phenomena allows for the calculation of the Lorentz force and the Joule heating, that are imported into the thermo-mechanical problem. Maxwell's equations are solved by FEM[3] for solid conductors, coupled with Boundary Elements (BEM)[4] for the surrounding vacuum, based on discrete differential forms of Nedelec-like elements[5]. When the FEM simulation has reached the transition to the plasma phase, its output parameters are input to the 3D resistive MHD code PLUTO to study the plasma expansion dynamics. In particular, the results of the density, displacement and velocity from the multi-physics LS-DYNA analysis are coupled to the single fluid 3D MHD modelling. PLUTO is a finite-volume/finite difference, shock-capturing code designed to integrate a system of conservation laws[6,7]. The workflow of the simulation is schematically described in Supplementary Fig. 1.

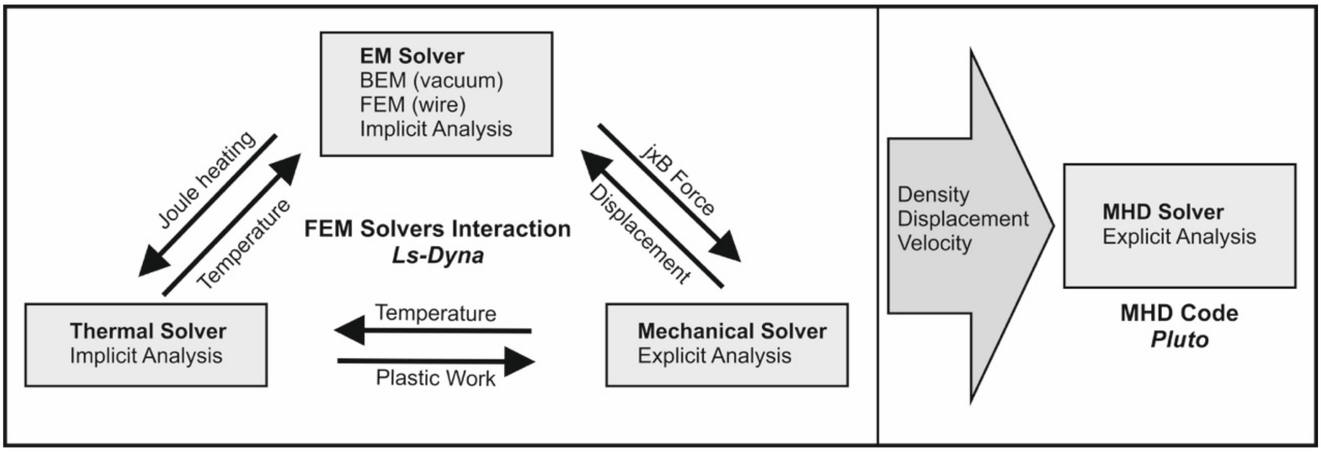

Supplementary Figure 1 | Workflow of the simulation methods. Coupling of the Finite Element Method (FEM) and Magneto-Hydrodynamic (MHD) numerical schemes.

Supplementary Note 1.1. | FEM modelling

The Lagrangian FEM model combines the electromagnetic, thermal and structural equations in a single coupled multiphysics simulation able to compute the transition of the metal wire from the solid to the plasma phase. The electromagnetic fields are computed by solving the Maxwell equations, with the Eddy-current (induction-diffusion) approximation. FEM is used for modelling the conductors coupled with Boundary Element Method (BEM) for the surrounding vacuum. When the electromagnetic fields are computed, the Lorentz force $\mathbf{F}=\mathbf{j}\times\mathbf{B}$, where $\mathbf{j}$ is the current density and $\mathbf{B}$ the magnetic field induction, is evaluated at the element nodes and added to the mechanical FEM solver that returns the deformation of the wire. The computed geometry is used for the calculation of the EM field evolution. The Joule heating power term (j^2/σ, where σ is the electrical conductivity) is added to the thermal solver and the temperature is updated.

Maxwell equations written in a quasi-stationary approximation and complemented with Ohm's law have the form:

$$\nabla\times\mathbf{E} = -\frac{\partial\mathbf{B}}{\partial t};\ \nabla\times\mathbf{B} = \mu_0\mathbf{j} \quad (1)$$

$$\mathbf{j} = \sigma\mathbf{E} + \mathbf{j}_s \quad (2)$$

where **E** is the electric field strength, $\mathbf{j}_s$ is the source current density and μ_0 is the permeability of vacuum. The mechanical solver is used to solve the hydrodynamic equations (3-5) that describe the laws of conservation of mass, the Cauchy momentum and energy:

$$\frac{d\rho}{dt} + \rho\nabla\mathbf{v}=0 \quad (3)$$

$$\rho\frac{d\mathbf{v}}{dt} = \nabla\boldsymbol{\sigma}_{ij} + \mathbf{j}\times\mathbf{B} \quad (4)$$

$$\rho\frac{dE}{dt} = \boldsymbol{\sigma}_{ij}\nabla\ \mathbf{v} + \eta j^2 \quad (5)$$

where ρ is the density, **v** is the velocity, $\boldsymbol{\sigma}_{ij}$ is the stress tensor, E energy and η the electrical resistivity. The stress tensor is represented by the index notation $\boldsymbol{\sigma}_{ij}$, where the first index refers to the face of the element being considered, and the second index refers to the direction of the stress. In order to calculate the stress tensor, the strain rate tensor, which describes the rate of change of the deformation of a material, is calculated:

$$\dot{\varepsilon}_{ij} = \frac{1}{2}(\frac{\partial v_i}{\partial x_j} + \frac{\partial v_j}{\partial x_i}) \quad (6)$$

Stress changes are related to the strain rate through the shear modulus G:

$$\frac{\partial\mathbf{s}_{ij}}{\partial t} = 2G(\dot{\varepsilon}_{ij} - \delta_{ij}\dot{\varepsilon}_{ii}) \quad (7)$$

where $\mathbf{s}_{ij}$ is the deviatoric stress tensor, the stress tensor without the contribution from hydrodynamic pressure and δ_{ij} is the Kronecker delta function.

Moreover, the thermal equation:

$$\rho c_p \frac{dT}{dt} = \nabla(k\nabla T) + \eta j^2 - L_{\mathrm{m}} \quad (8)$$

is solved by the thermal solver. c_p is the heat capacity, T temperature, k thermal conductivity and L_{m} latent heat of melting.

The dimensions of the wire in our simulations correspond to that of the real metal wire targets in the experiments. In Supplementary Fig. 2 a schematic of the 3D FEM model is depicted with an inset of a close view to its cross-section. As presented, hexahedral 8-node elements with one-point integration are chosen to carry out the FEM simulation. A small element size is used in the radial direction in order to accurately simulate the dynamic phase change of the metallic material both in the region of the skin depth and in the core region, where large compressive stresses are observed. A small element size is also used in the longitudinal direction in order to resolve the growth of the instabilities. The total number of elements for a 15 mm wire with a 0.15 mm radius was determined from different mesh discretization test cases to produce a mesh independent solution. Approximately 140000 elements were used (5 μm element length along radial direction and 50 μm along vertical direction) with 14000 BEM faces (shell elements) modelling the wire exterior. Test cases with high discretization densities in the mesh along the longitudinal axis direction of the wire were used so as to eliminate the possibility of numerical errors in the results. By increasing the finite elements number, along the longitudinal direction, we optimised the longitudinal discretization at 50 μm per element. For this element size, the wavelength of the observed instabilities remains the same in the range of 200 to 250 μm. With respect to the boundary conditions, the ends of the wire are at constant environmental temperature of 27 $^{\circ}$C. The initial temperature of the simulation domain is also considered to be 27 $^{\circ}$C and the wire is initially considered to be non-deformed. The loading source term is the pulsed current, as measured and recorded during the real experiments, with a peak of 40 kA and a rise time of 60 ns. The current waveform, as depicted in Figure 1 of the paper, is applied to the ends of the wire. The coupled multiphysics time-dependent problem is solved with an incremental time step of 0.1 ns for all solvers, in order to satisfy the conditions for time step control[1].

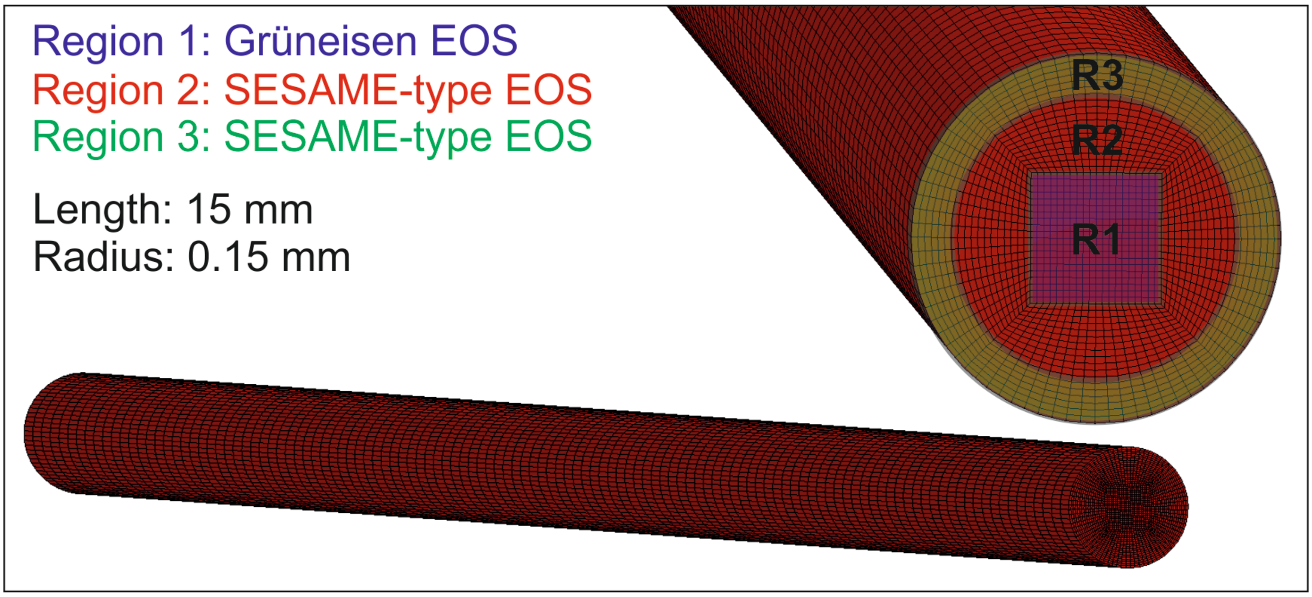

Supplementary Figure 2 | Model mesh and geometry. 3D FEM model of the wire and zoomed view of the cross-section and the Equations Of State (EOS) regions.

The material properties used in the simulations are based on the analytical Gruneisen[8] and tabular multiphase Equations Of State (EOS)[9]. These are coupled with a Johnson-Cook[10] strength material model for the study of the elastoplastic effects. The Burgess[11] EOS is used to determine the electrical resistivity as a function of temperature and density. Temperature dependent properties of the thermal expansion, thermal conductivity and specific heat, as well as the latent heat of melting are also taken into account. In the skin depth region, the pressure and temperature increase rapidly and the material reaches the melting or vaporization temperatures faster than the remaining part of the wire. Higher temperatures and lower densities are the main characteristics of this part of the wire. In contrast, the core region of the wire is characterized by high values of plastic strain, strain-rate, lower temperatures, and higher densities. Due to the wide ranges of temperature and density that have to be considered for the complete description of all the states of the matter, a SESAME-type tabular EOS is used because the Grüneisen equation has limitations in the expanded vapour zone. The Copper (Cu) EOS[9] is multi-phase and allows for the description of all the states of the matter: solid, liquid, gas, plasma and their transitions. In the initial phase, the Grüneisen EOS is used for the whole volume of the wire. The cross-section of the wire, as shown in Supplementary Fig. 2, is divided into three regions as defined by the feedback of preliminary numerical simulation using the

Grüneisen EOS. In region 1, where high stresses exist and temperature gradients are not very high, the Grüneisen EOS is used. The SESAME-type EOS for Cu is used for the regions 2 and 3 that correspond to the skin depth region. In these regions, the limits of the variables of temperature, density and pressure when the multiphase EOS is used were also defined by the feedback of preliminary numerical simulation using the Grüneisen EOS. The SESAME-type EOS tabular data of pressure in relation to energy[1] are interpolated for each density by a polynomial of the form:

$$P = P_{\mathrm{C}}(\mu) + P_{\mathrm{T}}(\mu, E) = C_0 + C_1\mu + C_2\mu^2 + C_3\mu^3 + (C_4 + C_5\mu + C_6\mu^2)E \qquad (9)$$

where C_0 to C_6 unknown set of parameters. A plot is created of the function $P=P(E)$ for each density of the tabular data and the slope is evaluated. The slopes are then fitted with a second order polynomial to obtain the coefficients (C_4 to C_6) of the thermal part $P_{\mathrm{T}}(\mu,E)$, where $\mu=(\rho/\rho_0)-1$ (ρ_0 is the reference density of the non-deformed state). The curve P-μ at the lower temperature of the tabular data is interpolated to obtain the coefficients of the cold curve $P_{\mathrm{C}}(\mu)$[12]. The parameters used in regions 2 and 3 are summarized in Supplementary Table 1.

	C_0 (Pa)	C_1 (Pa)	C_2 (Pa)	C_3 (Pa)	C_4	C_5	C_6
**Region 2**	-2.176×10^8	1.113×10^{11}	3.019×10^{11}	2.088×10^{11}	1.076	1.667	-0.039
**Region 3**					2	4.145	2.2

Supplementary Table 1 | Multiphase Equation Of State parameters

Supplementary Note 1.2. | MHD modelling

We study the plasma expansion dynamics using the resistive Eulerian MHD code PLUTO. The results of the density, displacement and velocity from the multi-physics LS-DYNA analysis are coupled to the 3D MHD modelling.

PLUTO is a finite-volume/finite difference, shock-capturing code[6,7,13] which solves the following system of the conservation laws of continuity, momentum, energy as well as the Faraday's law:

$$\frac{\partial \rho}{\partial t} + \nabla(\rho \mathbf{v}) = 0$$

$$\frac{\partial \mathbf{m}}{\partial t} + \nabla(\mathbf{mv} - \mathbf{BB} + I p_t) = 0$$

$$\frac{\partial E}{\partial t} + \nabla[(E + \rho_t)\mathbf{v}) - \mathbf{B}(\mathbf{vB})] = -\nabla[(\eta \mathbf{j})\times\mathbf{B}] \tag{10}$$

$$\frac{\partial \mathbf{B}}{\partial t} + \nabla\times(-\mathbf{v}\times\mathbf{B}) = -\nabla\times(\eta \mathbf{j})$$

where $\mathbf{m}=\rho\mathbf{v}$ is the momentum density, p_t is the total pressure (magnetic and thermal). An ideal gas EOS is considered, as well as Spitzer resistivity[14]. A structured mesh approach for the solution of the system of conservation laws is adopted. An equivalent set of primitive variables (mass density, velocity, pressure and magnetic induction) is used for assigning initial and boundary conditions. Flow quantities are discretized on a logically rectangular computational grid enclosed by a boundary and augmented with guard cells or ghost points in order to implement boundary conditions on a given computational stencil. Numerical integration of the conservation law, is achieved through shock-capturing schemes using the Finite Volume (FV) formalism where volume averages evolve in time. These methods are comprised of three steps: an interpolation routine, followed by the solution of Riemann problems at zone edges and a final time evolution stage.

Three regions are initially considered; a cylindrical fluid region, a cylindrical plasma region, and a cylindrical vacuum region. With respect to the 3D grid creation, discretization that ranges from 4-8 μm is used in our model along the radial and azimuthal direction, while a discretization of 2-4 μm is used for the longitudinal direction. The whole grid domain has a number of cells that ranges from 3×10^6-12×10^6 as presented in Supplementary Fig. 3. Concerning the assigned boundary conditions, the physical ghost zones of the computational domain outflow conditions are specified and a zero gradient of the flow quantities across the boundary are considered.

The density distribution, the displacement and the velocity outputs from the LS DYNA simulation when plasma is formed is coupled in PLUTO MHD code. The observed displacement instabilities in LS DYNA are imported as initial seed in PLUTO and their evolution is traced. At

every solution time step a restart analysis in PLUTO is performed coupling the new outcome data (density, displacement and velocity) from LS-DYNA.

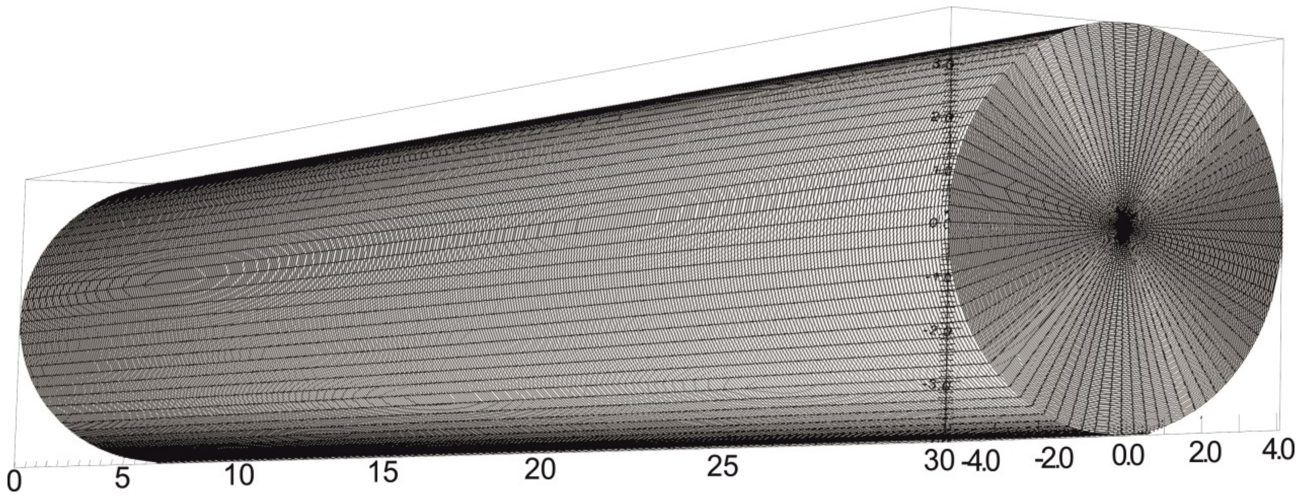

Supplementary Figure 3 | 3D grid. The structured grid of the 3D MHD model of the wire.

Supplementary Note 2 | Experimental validation methods

We use optical laser probing diagnostics such as shadowgraphy, schlieren, interferometry and Fraunhofer diffraction imaging, for diagnosing the spatio-temporal dynamics of the wire. The modified Fraunhofer diffraction optical probing method with high spatial resolution (~1 μm) is employed to determine the expansion of the wire before plasma formation. The combination of the above optical probing diagnostics also provides information for the early time history of the plasma formation including information on the phase change.

Experiments are performed using a Z-pinch, pulsed power device, that produces a peak current of 40 kA with a rise time of 60 ns. The Z-pinch device consists of a Marx bank of 600 J energy capacity, a water-filled pulse forming line (PFL) and a self-breaking SF_6 spark-gap switch. The Cu wire is placed in a vacuum chamber evacuated to a pressure of 10^{-4} mbar. The wire is fixed by soldering it to conical shaped Cu electrodes. A V-dot probe measures the derivative of the voltage at the PFL and a Rogowski groove measures the derivative of the current passing through the wire. The signals are recorded using a fast digital oscilloscope. The second harmonic of a SBS-compressed Nd:YAG, Q-switch laser with 150 ps pulse duration, is used for the optical probing diagnostics. This

enables sub-nanosecond resolution time resolved measurements of the wire's dynamics. In Supplementary Fig. 4 the electro-optical diagram of the experiment is presented.

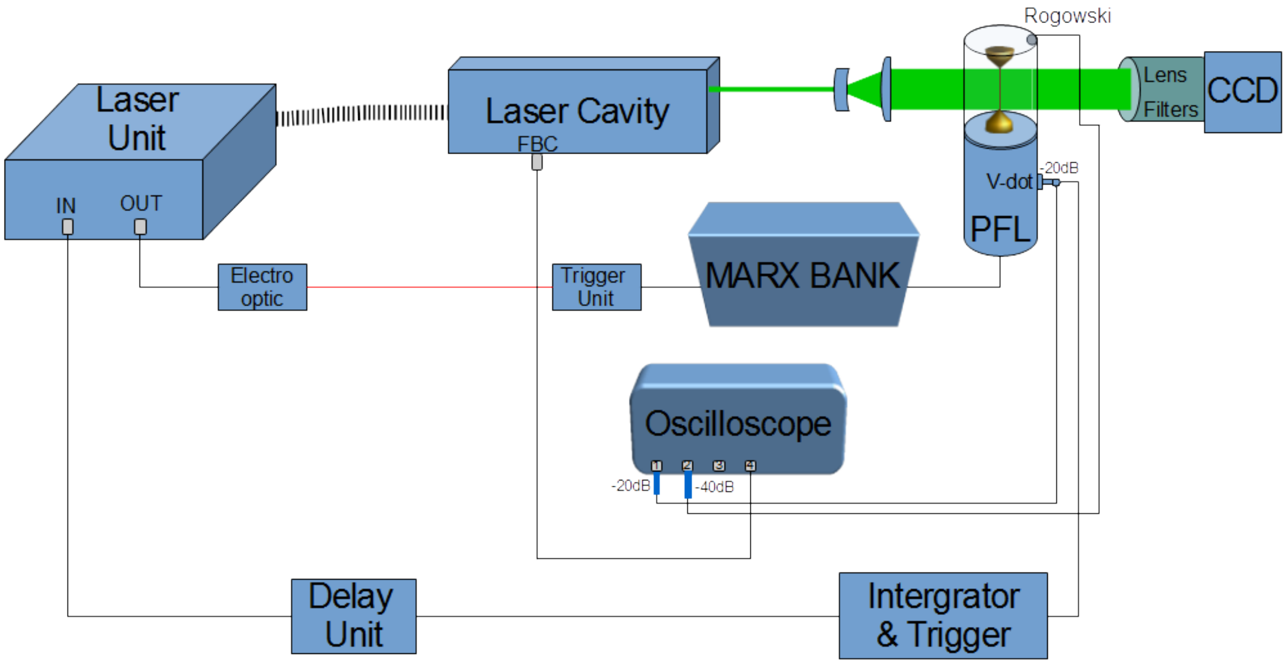

Supplementary Figure 4 | Electro-optic diagram of the experiment. Trigger signal from the laser control unit triggers the MARX bank, which provides a negative polarity high voltage output to PFL. When the voltage becomes equal to the breakdown voltage of the spark-gap switch, the current begins to flow through the wire to the anode. The V-dot output triggers the laser output while the delay unit defines the time delays used for the image capturing.

Shadowgraphy is used for the evaluation of the plasma instabilities along the wire. A Mach-Zehnder interferometer in finite-fringe mode is also used for plasma density measurements. For the schlieren imaging, a knife-edge is used at an orientation parallel to the wire at the focal length of the imaging lens so that is possible to distinguish the light deviation caused by plasma domination from that caused by neutral Cu vapors[15]. The formation of coronal plasma is determined from the bright light that appears on the same side of the wire as that where the knife-edge is placed[16]. In Supplementary Fig. 5 the set-up for the optical laser probing diagnostics is presented.

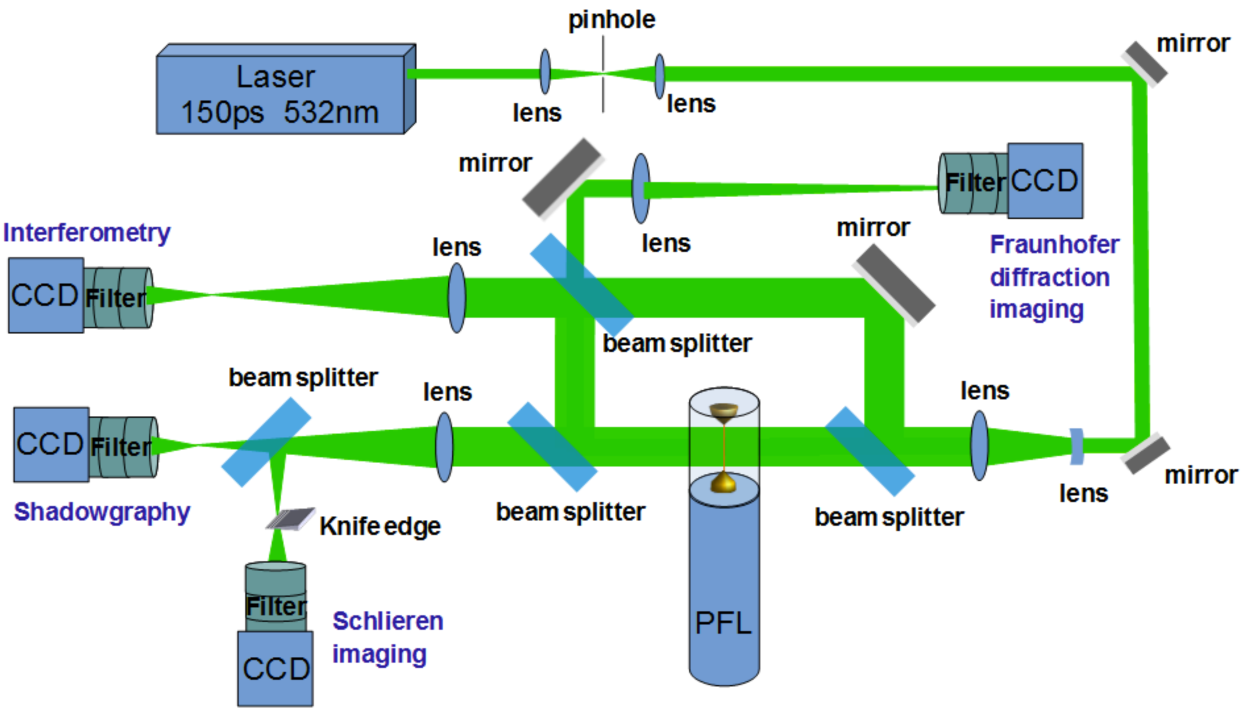

Supplementary Figure 5 | Experimental set-up. Optical laser probing diagnostics: Shadowgraphy, schlieren, interferometry and Fraunhofer diffraction imaging.

Supplementary Note 3 | Additional data

Supplementary Note 3.1. | Before plasma creation

As mentioned above, a modified Fraunhofer diffraction diagnostic is used to investigate the change in the wire's diameter before plasma formation. It is operated so that the 150 ps, 532 nm, laser beam which probes the wire forms the Fraunhofer diffraction pattern at the focal distance of the lens. The diffraction pattern is recorded on a CCD camera. The focal spot of the laser beam is shifted just out of the CCD's frame to reveal the second and higher order fringes at the image. The recorded diffraction image is compared to the reference image, captured before the shot to reveal the expansion of the wire. The diameter d, of the wire, is calculated by fitting a $sinc^2(x)$ function since the diffraction intensity is proportional to $sinc^2(\pi ds/f\lambda)$, where f is the focal length of the lens, λ is the wavelength of the laser and s is the real diffraction pattern length on the CCD[17]. Although the simplified expression gives a systematic overestimation of the wire's diameter, measurements

comparative to its initial value are very accurate, i.e., a small expansion can be measured with very high accuracy[17-19]. Supplementary Fig. 6 shows the lineout along the axis of the diffraction pattern at 140 ns from the current start, just before gas formation, compared to the reference (wire diameter before shot). The function and the results (errors are in the parenthesis) are shown in the inset. The measured diameter compared to the initial measurement shows an expansion of 7.4 (±0.8) μm. At that time the simulation gives an expansion of 7 μm. An average radial expansion rate of 65 (±1) m s^{-1} is experimentally measured while simulation results give a radial expansion rate of 60 m s^{-1}.

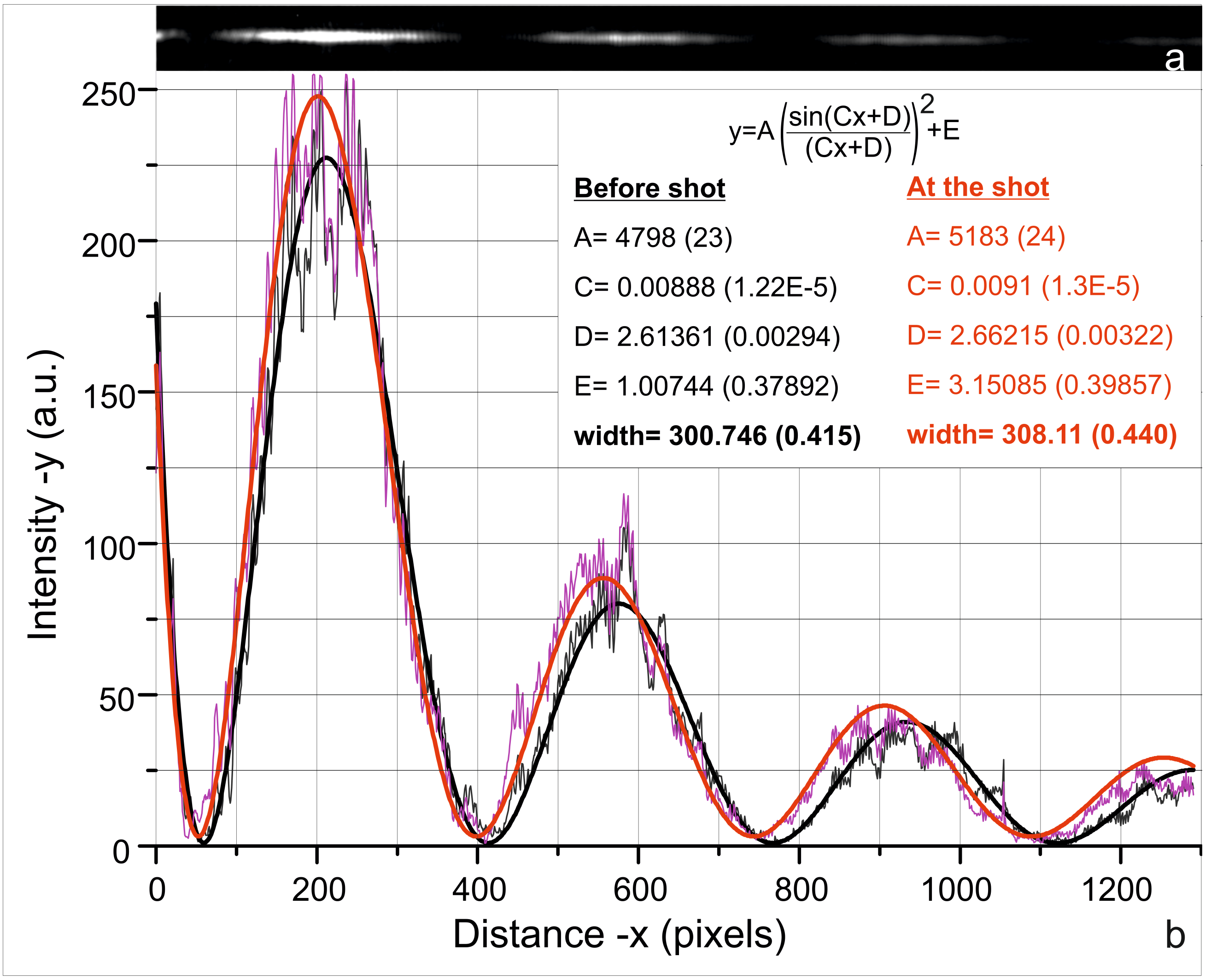

Supplementary Figure 6 | Fraunhofer diffraction method analysis. Laser probing diffraction pattern image (a) and the lineout intensity plot along the axis of the fringes (b) at 140 ns from the current start and at a time before the shot.

Simulation results from LS-DYNA show a significant deformation at the ends of the wire before plasma formation. In Supplementary Fig. 7 the behaviour at the end of the wire is depicted at 100 ns (Supplementary Fig. 7b). The initial un-deformed target before the interaction is also shown

(Supplementary Fig. 7a). The physical parameter of the plastic strain is shown in the longitudinal cross-section of the deformed geometry of the wire. This behaviour agrees with the experimental results where plasma usually appears first at the bottom of the wire where cathode is. An interferometric image is presented in Supplementary Fig. 7c with a zoomed detail at wire's end (cathode), where the shift of the fringes indicates the existence of vapour before plasma formation. In addition a larger diameter of the wire is shown at the cathode addressing the same behaviour as in the simulation.

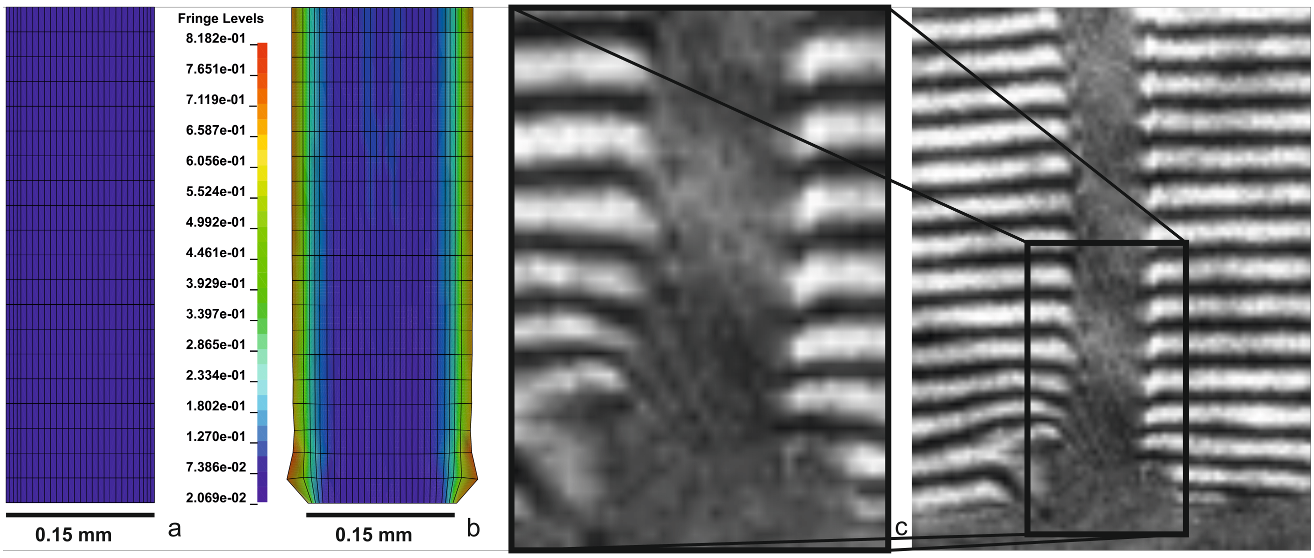

Supplementary Figure 7 | Matter's behaviour at the end of the wire. Computed deformation (b) of the created instabilities at the end of the wire, at 100 ns from current start. The interferometric image (c) is recorded just before plasma formation.

Supplementary 3.2. | In the plasma regime

In Supplementary Fig. 8 shadowgraphy, schlieren and interferometry images additional to the main manuscript are presented at 210 and 240 ns. The corresponding reference pre-shot images for each case are also shown. In the reference schlieren images the light along both edges of the wire is due to diffraction. After the shot, the bright light on the left and the darkening on the right side of the wire reveal the existence of coronal plasma (a knife edge parallel to the wire's axis was placed as a schlieren stop[16]). The fringes on the interferograms are shifted due to the plasma formation. At 210 ns the fringes are aligned transversely to wire's axis while at 240 ns the fringes are aligned parallel to the wire. The first mode is convenient for the evaluation of the radial expansion and the electron

density of the plasma, while the second one allows for the measurement of the wavelength of the plasma instabilities along the wire.

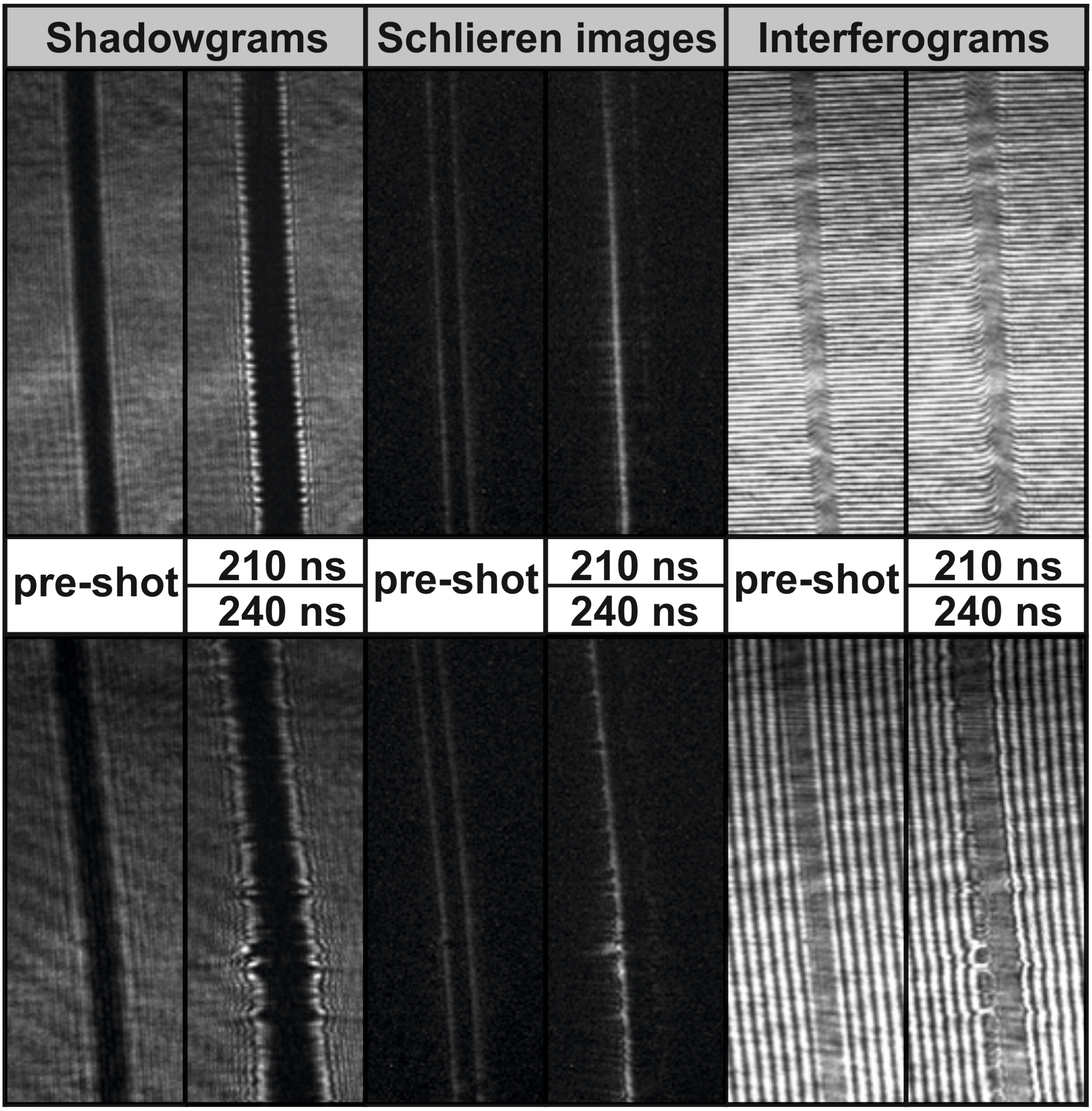

Supplementary Figure 8 | Plasma diagnostics. Experimental shadowgraphy, schlieren and interferometry images at 210 and 240 ns and their corresponding reference images.

The dominant average axial wavelength of instabilities in the coronal plasma 210 ns is measured to be 270 μm and the simulation computes it to be 255 μm, while 30 ns later is measured to be 600 μm and the simulation computes it to be 575 μm. The excellent agreement with the simulation for the whole spatiotemporal dynamics of the observed plasma evolution validates the

developed multiphysics model.

Here, we present resulting data of our MHD simulation studies - uncoupled from the thermo-elasto-plastic phase of the target – where the MHD is initiated by an artificial externally seeded perturbation function either sinusoidal, random or multispectrum periodic. These data are compared with those obtained when the real thermo-elasto-plastic seed is used as initial seed. Supplementary Figs 9 and 10 present plasma density contour plots at two different times showing the spatial distribution dynamics of the plasma density. For comparison, Supplementary Figs 9(a) and 10(a) present results when the real thermo-elasto-plastic seed is used as initial perturbation. It is shown that only when the real thermo-elasto-plastic seed is used the simulation results match the experiments. All other results show different instability structure that differs significantly from the experiments. These results clearly demonstrate how crucial is to consider the real intrinsic physical properties of the target when studying plasma instabilities.

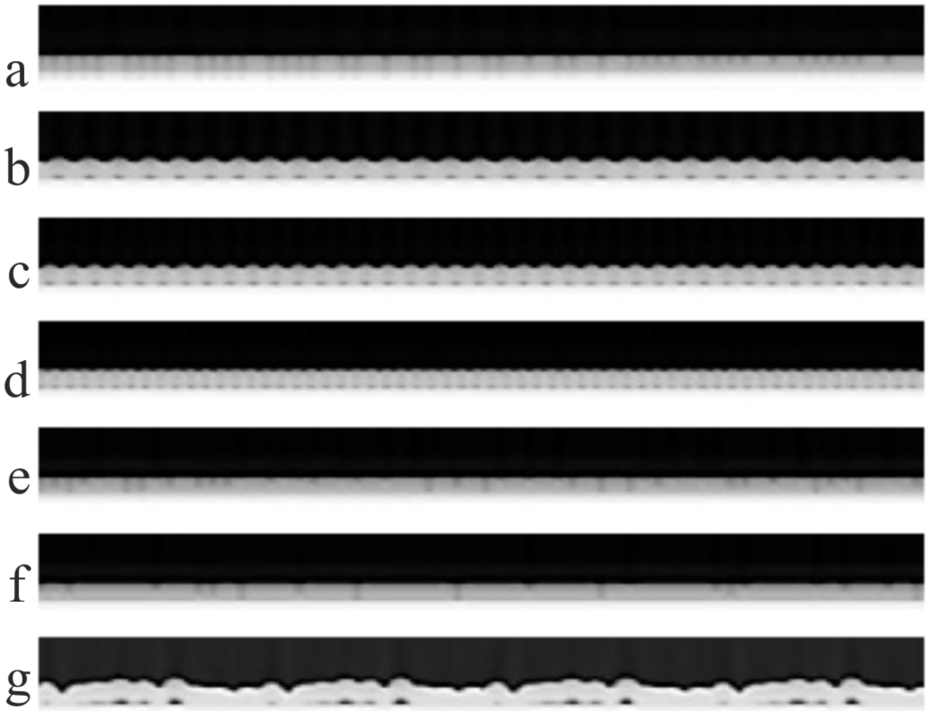

Supplementary Figure 9 | Plasma density contours at 210 ns from the current start. (a): initial perturbation seed is produced by the real thermo-elasto-plastic dynamics of the material (our study), (b, c, d): the seed is generated by an artificial sinusoidal function with 1.5 larger (b), equal (c) and 1.5 smaller (d) wavelength but same amplitude as in (a), are used respectively as initial seed. (e, f, g): random seed perturbation functions (e, f) and a multispectrum periodic function (g) are used as initial seed respectively. Indicatively the function in (g) is: cos(x)+0.5cos(3x+23)+cos(5x-0.4)+0.5cos(7x+2.09)+0.5cos(9x-3) and is plotted in Supplementary Fig. 11.

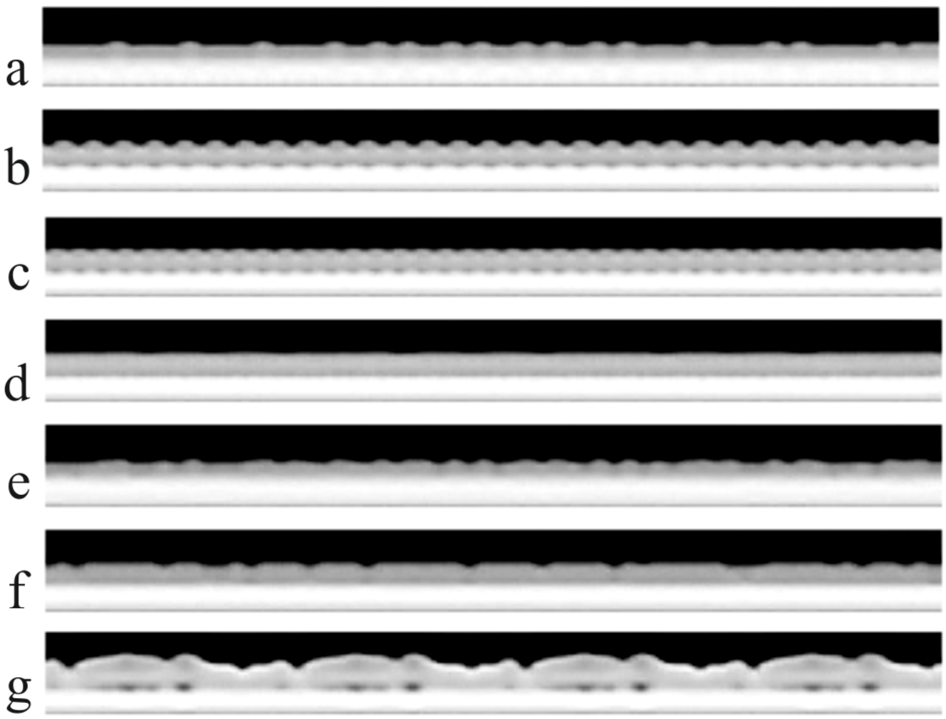

Supplementary Figure 10 | Plasma density contours at 240 ns from the current start. (a): initial perturbation seed is produced by the real thermo-elasto-plastic dynamics of the material (our study), (b, c, d): the seed is generated by an artificial sinusoidal function with 1.5 larger (b), equal (c) and 1.5 smaller (d) wavelength but same amplitude as in (a), are used respectively as initial seed. (e, f, g): random seed perturbation functions (e, f) and a multispectrum periodic function (g) are used as initial seed respectively.

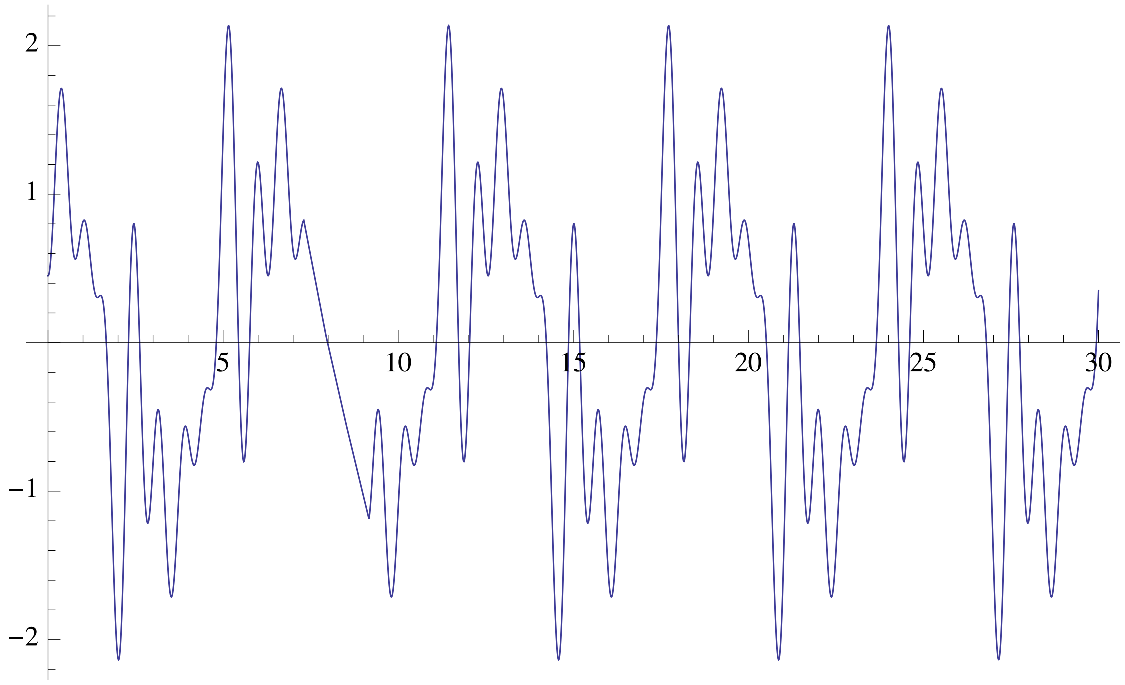

Supplementary Figure 11 | Multispectrum periodic function. Plotted function: cos(x)+0.5cos(3x+23)+cos(5x-0.4)+0.5cos(7x+2.09)+0.5cos(9x-3) which serves as initial density perturbation in Supplementary Figs 9g and 10g.

Supplementary Note 4. | Future research on the seeding mechanisms of plasma instabilities

As stated in the main manuscript, our proof-of-principle study is not aiming to discuss in details aspects of plasma instabilities such as the kind of instability, the growth rates, the wavelengths etc., but to present the physics that needs to be considered at the very first moments within the Joule heating taking into account the intrinsic real physical properties of the target. Adaptation of our multiphysics methodology to known instabilities such as, for example, the electrothermal instability (ETI) – which begins to grow immediately after the heating source is applied onto the solid target – is the purpose of future work. However, here we give an introductory discussion identifying the frame for the specialization of this work for the study/modification of prompt instabilities such as the electrothermal instability (ETI) in the linear or non-linear magnetic diffusion regime.

When a material with temperature-depended electrical resistivity is Ohmically heated it can be subject to the development of ETIs, where a temperature perturbation due to Joule heating can grow in time and space[20-36]. An ETI is a prompt instability, begins to grow immediately after the heating source is applied and persists as the outer part of the target expands rapidly. If $d\eta/dT > 0$ (where η is the electrical resistivity and T the temperature of the material) thermal instabilities give rise to layered structures called strata, which are developed normal to the current flow[25-27,31-34,37-40]. This type of instability dominates at current densities greater than 10^7 A cm^{-2}. Sometimes this instability is also referred to as overheat instability[32,41]. On the other hand when $d\eta/dT < 0$ as for example in the Spitzer resistivity of a plasma, ETIs give rise to filamentation of the current flow into channels[21,35]. Furthermore, it is very well known that during an electrical explosion the material undergoes all phases of matter from solid to plasma. The ETI has become the subject of active research due to its importance as a potential seed mechanism for the Magneto-Rayleigh-Taylor (MRT) instability growth. The growing ETI gives rise to pressure nonuniformities at the surface

acting as the potential seed for the MRT instability, which appears when the magnetic pressure increases up to the point able to oppose the rapid expansion of the material. At this point, the MRT instability – also known as flute instability – begins to grow seeded by the ETI of the previous phase[43].

Other studies have pointed out the importance on the development of ETI of the nonlinear magnetic diffusion into the target during electrical explosions [26,42]. During an electrical explosion of a conductor at the skin effect mode the formation of a dense low temperature plasma at the surface of the conductor is accompanied by the generation and propagation of a shock wave and a nonlinear magnetic diffusion wave (NMDW). For the majority of metals the magnetic induction threshold above which a NMDW can take place is a few hundreds of kGauss. In this case the NMDW propagates in the conductor together with the shock wave generated at the surface. As time progresses, the magnetic diffusion rate increases due to the increase of the electrical resistivity of the metal. It was shown that when a NMDW propagates through a conductor the long wavelength modes of the ETI are suppressed and that the short wave modes of ETI can be unstable[26,42-43]. Recent studies with the inclusion of heat conduction, shock-wave propagation as well as viscous damping, also exposed the crucial role of the ETI to the growth of the MRT instability[26,42-43]. It was also proposed that in pulsed power magnetic liners the initial seed of the MRT instability growth is the electrothermal instability[44] and not the surface roughness. Experiments showed results on the mitigation of the growth of the MRT instability on metallic rods and liners when dielectric coatings were applied at the surface to mitigate the ETI seed[33-34].

Therefore, it is clear and widely recognized by the scientific community[21,42,45] that the complexity of such a multiphysics problem builds serious difficulty for the simulations on magnetically driven and laser ICF implosions, to successfully incorporate the realistic physical initial conditions of the targets. Up-to-date all studies were limited to the coupling of properties of the solid, among others the resistivity, the heat conduction, the magnetic diffusion, the viscous damping and

the stress tensors, to the system of the MHD equations. These studies treat the target at its initial condition as an MHD fluid having a solid density, including terms to comprise some of the physical properties of the solid material.

Early stage results of the application of our approach for the study of the ETI for our experimental conditions show that the growth rates of this instability - which serves as the seed for the MRT instability - is at least one order of magnitude larger than those found in the literature. This difference is due to our treatment of the target as an electro-thermo-mechanical system, that takes into account the target's mechanical and physical properties. The inclusion of the material's mechanical properties modifies the ETI into an instability, which for future reference we choose to call it electro-thermo-mechanical (ETM) instability.

Supplementary References

[1] Hallquist, J. O. LS-dyna Theory Manual, LSTC, Livermore, 2006.

[2] Caldichoury, I & L'Eplattenier, P. EM theory manual, LSTC, Livermore, 2012.

[3] Jin, J. *The Finite Element Method in Electromagnetics*, (Wiley, 1993).

[4] Shen, J. *Computational Electromagnetics Using Boundary Elements, Advances In Modelling Eddy Currents, Topics in Engineering* Vol. 24 (eds Brebbia, C. A. & Connor, J. J.) (Computational Mechanics Publications, 1995).

[5] Nedelec, J. C. A New Family of Mixed Finite Elements in R3, *Num. Math.* **50**, 57-81 (1986).

[6] Mignone, A. *et al*. PLUTO: A Numerical code for computational Astrophysics, *Astrophys. J., Suppl.* **170**, 228-242 (2007).

[7] Mignone, A. *et al*. The PLUTO Code for Adaptive Mesh Computations in Astrophysical Fluid Dynamics, *Astrophys. J., Suppl.* **198**, 7 (2012).

[8] Nagayama, K. *Introduction to the Gruneisen Equation of State and Shock Thermodynamics* (Kindle Edition, 2011).

[9] Kerley, G. I. Equation of State for Copper and Lead, KTS02-1, 2002.

[10] Johnson, G. R. & Cook, W. H. Fracture characteristics of three metals subjected to various strains, strain rates, temperatures and pressures, *Eng. Fract. Mech.* **21**, 31-48 (1985).

[11] Burgess, T. J. Electrical resistivity model of metals, 4th International Conference on Megagauss Magnetic-Field Generation and Related Topics, Santa Fe, NM, USA, 1986.

[12] Scapin, M., Peroni, L. & Dallocchio, A. in *Numerical Modeling of Materials Under Extreme Conditions* (eds Bonora, N. & Brown, E.) p. 87-106 (Springer, 2014).

[13] PLUTO v. 4.1, User's guide, 2014.

[14] Spitzer, L. & Haem, R. Transport Phenomena in a Completely Ionized Gas, *Phys. Rev.* **89**, 977-981 (1953).

[15] Huddlestone, R. H. & Leonard, S. L. *Plasma diagnostic techniques*, (Academic Press, 1965).

[16] Tkachenko, S. I. *et al.* Distribution of matter in current-carrying plasma and dense core of the discharge channel formed upon electrical wire explosion, *Plasma Phys. Rep.* **35**, 734-753 (2009).

[17] Lebrun, D. *et al.* Enhancement of wire diameter measurements: comparison between Fraunhofer diffraction and Lorenz-Mie theory, *Opt. Eng.* **35**, 946-950 (1996).

[18] Sanchez-Brea, L. M. & Salgado-Remacha, F. J. Three-dimensional diffraction of a thin metallic cylinder illuminated in conical incidence: application to diameter estimation, *Appl. Opt.* **47**, 4804-4811 (2008).

[19] Zhu H. *et al.* Measurement of thermal energy coupling to metallic materials in millisecond laser based on optical diffraction, *Opt. Laser Technol.* **90**, 84-89 (2017).

[20] Cap, F. F. *Handbook on Plasma Instabilities* Vol. 1-3 (Academic Press, 1976, 1978, 1982.

[21] Haines, M. G. A review of the dense Z-pinch, *Plasma Phys. Control. Fusion* **53**, 093001 (2011).

[22] Fansler, K. S. & Shear, D. D. in *Exploding Wires*, (eds Chace, W. G. & Moor, H. K.) p. 185 (Springer 1968).

[23] Kadomtsev, B. B. in *Reviews of Plasma Physics*, (ed M. A. Leontovich) (Consultants Bureau, 1980).

[24] Epelbaum, Y. G. Thermal and hydromagnetic instability in a current-carrying liquid metal cylinder, *Sov. Phys. Tech. Phys.* **29**, 293 (1984).

[25] Oreshkin, V. I. *et al.* Wire explosion in vacuum: Simulation of a striation appearance, *Phys. Plasmas* **11**, 4771 (2004).

[26] Oreshkin, V. I. & Chaikovsky, S. A. Stability of a nonlinear magnetic field diffusion wave, *Phys. Plasmas* **19**, 022706 (2012).

[27] Rousskikh, A. G. *et al.* Study of the strata formation during the explosion of a wire in vacuum, *Phys. Plasmas* **15**, 102706 (2008).

[28] Anisimov, S. I. & Zeldovich, Ya. B. Rayleigh–Taylor instability of the interface between the detonation products and a gas in a spherical explosion, *Sov. Tech. Phys. Lett.* **3**, 445-446 (1977).

[29] Haan, S.W. *et al.* Point design targets, specifications, and requirements for the 2010 ignition campaign on the National Ignition Facility, *Phys. Plasmas* **18**, 051001 (2011).

[30] Peterson, K. J. *et al.* Simulations of electrothermal instability growth in solid aluminum rods, *Phys. Plasmas* **20**, 056305 (2013).

[31] Oreshkin, V. I. Thermal instability during an electrical wire explosion, *Phys. Plasmas* **15**, 092103 (2008).

[32] Oreshkin, V. I. Overheat Instabilities during the Electrical Explosion Wires, *Tech. Phys. Lett.* **35**, 36-39 (2009).

[33] Peterson, K. J. *et al.* Electrothermal Instability Mitigation by Using Thick Dielectric Coatings on Magnetically Imploded Conductors, *Phys. Rev. Lett.* **112**, 135002 (2014).

[34] Awe, T. J. et *al.* Experimental Demonstration of the Stabilizing Effect of Dielectric Coatings on Magnetically Accelerated Imploding Metallic Liners, *Phys. Rev. Lett.* **116**, 065001 (2016).

[35] Haines, M. G. Thermal Instability and Magnetic Field Generated by Large Heat Flow in a Plasma, Especially under Laser-Fusion Conditions, *Phys. Rev. Lett.* **47**, 917-920 (1981).

[36] Craxton, R. S. *et al*. Direct-drive inertial confinement fusion: A review, *Phys. Plasmas* **22**, 110501 (2015).

[37] Braginsky, S. I. in *Reviews of Plasma Physics*, (ed. Leontovich, M. A.) (Consultants Bureau, 1980).

[38] Raizer, Yu. P. *Gas Discharge Physics* (Springer, 1997).

[39] Sinars, D. B. *et al*. Experiments measuring the initial energy deposition, expansion rates and morphology of exploding wires with about 1 kA/wire, *Phys. Plasmas* **8**, 216 (2001).

[40] Sarkisov, G. S., Sasorov, P. V., Struve, K. W. & McDaniel, D. H. State of the metal core in nanosecond exploding wires and related phenomena, *J. Appl. Phys.* **93**, 1674 (2004).

[41] Haines, M. G. An electron thermal instability in a resistive non-equilibrium fully ionised plasma, *J. Plasma Phys.* **12**, 1-14 (1974).

[42] Chaikovsky, S. A. *et al*. Experimental study of the nonlinear diffusion of a magnetic field and skin explosion of cylindrical conductors, *Phys. Plasmas* **22**, 112704 (2015).

[43] Oreshkin, V. I. *et al*. MHD instabilities developing in a conductor exploding in the skin effect mode, *Phys. Plasmas* **23**, 122107 (2016).

[44] Peterson, K. J. *et al*. Electrothermal instability growth in magnetically driven pulsed power liners, *Phys. Plasmas* **19**, 092701 (2012).

[45] Awe, T. J. *et al*. On the Evolution From Micrometer-Scale Inhomogeneity to Global Overheated Structure During the Intense Joule Heating of a z-Pinch Rod, *IEEE Transactions on Plasma Science* **45**, 584-589 (2017).
